# Memory Impairment following Acute Tricyclic Antidepressants Overdose

**DOI:** 10.1155/2015/835786

**Published:** 2015-01-14

**Authors:** Nastaran Eizadi-Mood, Shahla Akouchekian, Ahmad Yaraghi, Mehrnazsadat Hakamian, Rasool Soltani, Ali Mohammad Sabzghabaee

**Affiliations:** ^1^Isfahan Clinical Toxicology Research Center, Isfahan University of Medical Sciences, Isfahan, Iran; ^2^Behavioral Sciences Research Center, Isfahan University of Medical Sciences, Isfahan, Iran; ^3^Department of Anesthesiology and Critical Care, School of Medicine, Isfahan University of Medical Sciences, Isfahan, Iran; ^4^Department of Clinical Toxicology, Noor and Ali-Asghar (PBUH) University Hospital, Isfahan University of Medical Sciences, Isfahan, Iran; ^5^Department of Clinical Pharmacy and Pharmacy Practice, School of Pharmacy and Pharmaceutical Sciences, Isfahan University of Medical Sciences, Isfahan, Iran

## Abstract

*Background*. Psychiatric consultation is necessary for all patients with intentional poisoning and its reliability depends on the proper function of patients' memory performance. This study aimed to determine the possible memory impairment following acute TCAs' poisoning. *Materials and Methods*. In this cross-sectional study, patients with acute TCAs poisoning were allocated to two groups of severe poisoning (with coma, seizures, cardiac arrhythmias, hypotension, and a wide QRS complex) and mild-to-moderate poisoning according to their clinical presentation at the time of hospital admission. All patients underwent memory performance test both immediately and 24 hours after their initial consciousness after admission, using Wechsler Memory Scale (WMS-IV). *Results*. During the study period, 67 TCA-poisoned patients (aged, 20–64 years) were evaluated, of which 67.2% were female. The mean memory scores of patients immediately and 24 hours after the initial consciousness were 31.43 ± 9.02 and 50.62 ± 9.12, respectively (*P* < 0.001). Twenty-four hours after the initial consciousness, memory score was statistically correlated with the amount of ingested drug and the intoxication severity. *Conclusion*. Following the recovery from somatic symptoms of acute TCA poisoning, patients may still suffer from memory impairment and it seems that this time is not suitable for performing a reliable psychiatric consultation.

## 1. Introduction

There are limited studies about memory impairment following drug overdose. From years ago it was found that following benzodiazepines overdose memory impairment will occur [1–3] which can remain for more than 24 hours after patient consciousness as anterograde memory impairment [2]. Memory impairment has also been reported after the overdose of some antipsychotic drugs like olanzapine [4].

In many parts of the world, tricyclic antidepressants (TCAs) are still used for the treatment of a variety of disorders including major depression, anxiety, migraine headache, and chronic pain [5, 6]. According to the wide availability of TCAs for the treatment of depressed patients, the prevalence of using these drugs for suicide attempt still remains high [7]. TCAs' poisoning is also the most frequent cause of drug-induced death in some countries [8, 9]. Many of the initial signs of TCA poisoning are those associated with the anticholinergic effects of these drugs including memory impairment [10]. Memory deficit following drug overdose is an important issue considering that all patients with intentional poisoning will undergo psychiatric consultation after symptoms resolution and before hospital discharge in order to evaluate the possible cause of suicide attempt. Hence, if TCAs' poisoning causes anterograde memory impairment, the validity of the psychiatric counseling would be questionable and patients may not get the psychiatric advisory content effectively at the time of their discharge. In this case, psychiatric counselor can provide the patients and his/her relatives with written information about arrangements made for further managing suicidality and treatment [11].

To the best of our knowledge there is no human study about memory impairment following TCAs' overdose and poisoning. In an animal study, intraperitoneal injection of imipramine caused memory impairment in rat via action on gamma aminobutyric acid type B receptors (GABA-B receptors) [12]. Also, another study showed that intraperitoneal injection of imipramine resulted in memory deficits in rat via mechanisms involving *α*_2_-adrenoceptors [13].

Since TCAs' poisoning is among the most dangerous single-drug overdoses in our referral poisoning emergencies department and many other similar poison management centers, this study aimed to determine possible memory impairment following acute TCAs' poisoning and also to detect any possible relationship between the memory deficit and TCAs' poisoning severity.

## 2. Methods

This was a cross-sectional study conducted in the Poisoning Emergency Department of Noor and Ali Asghar (PBUH) University hospital in Isfahan, Iran. The study protocol was approved by the Ethics Committee of the Isfahan University of Medical Sciences with the registration number of 384250. Patients aged 20 to 64 years who were referred to this department with the clinical manifestations of TCA poisoning (hypotension, arrhythmias, seizures, hyperthermia, delirium, ventricular or sinus tachycardia, urinary retention, myoclonus, wide QRS, mydriasis, and unconsciousness) were included in the study. Patients with a history of dementia (according to DSM-IV diagnostic criteria) or known chronic diseases affecting the cognitive function (e.g., hypothyroidism) were excluded from the study. Also, patients with mixed drug ingestion (including alcohol), cardiac and respiratory arrest, and those who did not have the necessary cooperation during the test were excluded.

Demographic characteristics as well as the type and dose of the ingested drug according to patients' history and the tablet samples provided by the relatives were recorded for all of the recruited patients. According to the clinical presentation, patients with inclusion criteria were divided into two groups of severe poisoning who presented with coma, seizures, cardiac arrhythmias, hypotension, and a wide QRS complex, and mild-to-moderate poisoning (patients without those aforementioned clinical conditions at the time of admission) and managed accordingly. Following resolution of poisoning symptoms, all patients underwent memory test in physically standard conditions both immediately and 24 hours after patients were awake and were able to communicate using Wechsler Memory Scale-fourth edition (WMS-IV) [14]. The WMS-IV is made up of a battery of 7 subtests. Four of the subtests have both an immediate and delayed recall conditions. The subtests include general cognitive screener, immediate and delayed logical memory (recall of details of a short story), immediate and delayed verbal paired associates (recalls of the second word of a pair after prompting with the first), immediate and delayed design memory (recall of both the content and spatial arrangement of images within a grid), immediate and delayed visual reproduction (replication of a line drawing after viewing it for ten seconds), spatial addition (reconstruction of two grid patterns in a single space), and symbol span (recollection of an increasing number of symbols in the order in which they were presented); the two later subtests are visual working memory tasks and are only administered once [14].

We used the 14th version of statistical package for social sciences (SPSS Inc., Chicago, IL, USA) for statistical analysis of our data. Pearson's correlation coefficients were used to detect any possible correlation of memory score with patient age and drug dosage. Spearman's correlation coefficient was calculated for detection of correlation of memory score with poisoning severity. To determine the difference between the mean of memory scores at the two tested times, paired-samples *t*-test was done. *P* < 0.05 was considered as significant.

## 3. Results

A total of 67 patients (45 females) aged 20–64 years (median, 24) who had a reliable history and clinical manifestations of TCAs' poisoning were studied (Table 1). Nortriptyline, Amitriptyline, and Imipramine poisoning were more frequent and 18 patients were previously using the drug for their medical illnesses. Of these patients, 42 were intoxicated with a total estimated dose of 100–500 mg and 18 with more than 1000 mg of the antidepressant drug.

The average memory scores of patients when they were awake and able to communicate and 24 hours after resolution of somatic symptoms were 31.43 ± 9.02 and 50.62 ± 9.12, respectively (*P* < 0.001). Table 1 shows the average memory scores of patients in terms of different variables at the two times of memory performance evaluation. As shown, at the time of initial consciousness, memory score had no correlation with patient gender, age, and severity of intoxication while it had a correlation with drug dosage. Twenty-four hours after the patients were awake, memory score was correlated with patient age, drug dosage, and the severity of intoxication and the memory status score of patients was improved close to the normal range.

## 4. Discussion

Our results show that 24 hours after resolution of somatic symptoms of TCAs' poisoning, the memory score of TCA-overdosed patients is significantly higher compared to the time when patients were first awakened and able to communicate. This finding indicates that although TCA-poisoned patients become conscious few hours after poisoning symptoms attenuation, they suffer memory impairment that will improve significantly during the next 24 hours. Therefore, despite the consciousness and orientation of the patient and the feasibility of his/her discharge from the poisoning ward, it seems that the psychological or psychiatric consultation at this time is less efficient compared to 24 hours later. On the other hand, the risk of further suicide attempts in patients who may have a history of depression or TCAs is accessible for them underscores the importance of an efficient psychiatric consultation in an ideal time of discharge from the hospital. Therefore the discharge time for these patients is debatable despite the stable medical and somatic conditions.

Effect of antidepressants on memory performance in depressed outpatients is studied elsewhere [15, 16]. But to the best of our knowledge, there is no study on memory impairment following TCAs overdose. In therapeutic doses, TCAs may cause memory impairment [17]. According to some other studies, the impairing effects of TCAs on memory are mainly related to the anticholinergic action of these drugs [18, 19]. Muscarinic receptors are the dominant cholinergic receptors in the brain being involved in memory and learning. TCAs (e.g., amitriptyline and trimipramine) are clearly more effective than some other antidepressant drugs, in blocking muscarinic receptors leading to memory dysfunction [20]. Animal studies have proposed other mechanisms of memory impairment by TCAs including GABA-ergic and *α*_2_-adrenergic activities [12, 13].

Our results show that there is a significant correlation between memory score at the time when TCAs poisoned patients were awake (able to communicate) and 24 hours later (when somatic symptoms are resolved). There is also a statistically significant correlation between the amount of drug ingested and memory score in both times of memory evaluation which could be due to the increased drug effects on muscarinic and probably GABA- and *α*_2_-adrenergic receptors especially in higher doses. In the study of Meador-Woodruff et al., 9 out of 16 patients with plasma TCA levels greater than 450 ng/mL developed cognitive impairment compared to only 1 out of 15 patients in the control group with plasma TCA levels of 150–450 ng/mL [21]. Such a relationship between memory score and drug dosage has also been reported for benzodiazepines [3, 22, 23]. A similar correlation is also observed between memory score and patients' age in our study which could be related to a probable decrease in liver and kidney function at higher ages and leading to the exacerbation of drugs' toxicological effects. Therefore, it may be more logical to implement psychological or psychiatric consultation after the first day of initial consciousness if the patient is poisoned with large doses of the TCA.

Our study had some limitations. First of all, if we could have confirmatory serum levels of TCAs or at least a qualitative screening test in our patients, this could increase the reliability of our results. Secondly, we had sampling from one referral hospital and although this sampling was done in a systematic way, it may lead to some concerns for the extrapolation of results. We also had some patients (*n* = 18) that were using TCAs for the treatment of depression and/or obsessive compulsive disorders before poisoning and this may be considered as a confounding factor for the results.

## 5. Conclusion

Following the recovery from the somatic symptoms of TCA overdose, patients may suffer from memory impairment and it seems that this time is not suitable for the psychiatric consultation. Further complementary studies are required for monitoring the memory status and the competence of TCA-poisoned patients for longer times (more than 24 hours) after initial recovery of the patients from the somatic symptoms. This may help to determine the optimum time for psychiatry consultation of these patients more precisely.

## Figures and Tables

**Table 1:** Memory score of TCA-poisoned patients in terms of different parameters at the time of initial consciousness^†^ and 24 hours afterward (mean ± SD) (*n* = 67).

Parameter	Category	*n*	Initial consciousness		24 hours after consciousness	
			Memory score	*P* value	Memory score	*P* value
Gender	Male	22	29.97 ± 9.40	0.36^*^	49.79 ± 9.89	0.60^*^
	Female	45	32.14 ± 8.83		51.03 ± 8.78	

Age range	20–24 yrs	45	32.80 ± 8.50	0.11^**^	52.48 ± 8.90	0.002^**^
	25–34 yrs	14	30.32 ± 9.60		47.85 ± 8.10	
	35–44 yrs	5	26.90 ± 6.0		47.50 ± 2.10	
	45–54 yrs	2	30.75 ± 8.83		49.0 ± 8.40	
	55–64 yrs	1	9.0 ± 0.0		24.50 ± 0.0	

Severity	Mild-to-moderate	52	32.31 ± 8.94	0.13^***^	46.30 ± 8.50	0.03^***^
	Severe	15	28.36 ± 8.93		51.85 ± 8.90	

Dosage range	100–500 mg	42	34.71 ± 7.40	<0.001^**^	53.48 ± 8.10	<0.001^**^
	501–1000 mg	7	34.37 ± 8.50		53.62 ± 8.50	
	>1000 mg	18	21.94 ± 5.80		42.10 ± 6.10	

Total		67	31.43 ± 9.02		50.62 ± 9.12	<0.001^****^

^†^When the patients were awake and were able to communicate after admission.

^*^Independent samples *t*-test; ^**^Pearson correlation test; ^***^Spearman correlation test; ^****^paired-samples *t*-test.
